# Safety and effectiveness of pembrolizumab monotherapy in Japanese patients with unresectable urothelial carcinoma: a nation-wide post-marketing surveillance

**DOI:** 10.1186/s12885-023-10930-2

**Published:** 2023-06-20

**Authors:** Hiroyuki Nishiyama, Yu Tanaka, Masahiro Hamada, Masahiko Ozaki, Toshihiko Minegishi, Yuichiro Ito, Shinichiroh Maekawa, Nobuyuki Yamamoto

**Affiliations:** 1 Department of Urology, Faculty of Medicine, University of Tsukuba, 1-1-1 Tennodai, Tsukuba, 305-8575 Japan; 2 Japan Pharmacovigilance, MSD K.K., Tokyo, 1-13-12 Kudan-kita, Chiyoda-ku, 102-8667 Japan; 3 Oncology Medical Affairs, MSD K.K., Tokyo, 1-13-12 Kudan-kita, Chiyoda-ku, 102-8667 Japan; 4 Respiratory Medicine and Medical Oncology, Wakayama Medical University, 811-1 Kimiidera, Wakayama, 641-8509 Japan

**Keywords:** Japanese patients, Pembrolizumab, Post-marketing surveillance, Unresectable urothelial carcinoma

## Abstract

**Background:**

This study was conducted to identify factors associated with the safety and effectiveness of pembrolizumab in Japanese patients with unresectable urothelial carcinoma and to confirm the real-world safety and effectiveness of pembrolizumab in Japanese patients.

**Methods:**

This multicenter, observational, post-marketing surveillance was conducted over a 1-year observation period starting at pembrolizumab initiation (200-mg pembrolizumab every 3 weeks); data were collected from case report forms (3 months and 1 year). Safety measures included treatment-related adverse events and adverse events of special interest (AEOSI). Effectiveness assessments included tumor response, objective response rate (ORR), and disease control rate (DCR).

**Results:**

Overall, 1293 patients were evaluated for safety and 1136 for effectiveness. At 12 months, the treatment-related adverse event incidence was 53.8% (*n* = 696) and that of AEOSI was 25.0% (*n* = 323). The most frequent AEOSI of any grade were endocrinological disorder (10.4%, *n* = 134), interstitial lung disease (ILD) (7.2%, *n* = 93), and hepatic function disorder (4.9%, *n* = 64). Multivariate analysis demonstrated that the risk of developing ILD was almost seven times greater (odds ratio 6.60) in patients with a comorbidity of ILD, and approximately twice as high in patients aged ≥ 65 years (odds ratio 2.24) and with smoking history (odds ratio 1.79). The ORR was 26.1% and the DCR was 50.7%. The ORR was 46.4% in patients with a Bellmunt risk score of 0 and decreased as the Bellmunt risk score increased.

**Conclusions:**

This post-marketing surveillance confirmed the safety and effectiveness of pembrolizumab in Japanese patients with unresectable urothelial carcinoma in the real-world setting.

**Supplementary Information:**

The online version contains supplementary material available at 10.1186/s12885-023-10930-2.

## Background

Urothelial carcinoma can arise from the renal pelvis, ureter, bladder, and proximal urethra; it is the most common histologic subtype of bladder cancer, accounting for approximately 95% of bladder cancer cases [1]. Bladder cancer is the tenth most common cancer worldwide [2], and the thirteenth most common cancer in Japan [3]. The age-standardized incidence and mortality rate is 5.6 and 1.9 per 100,000, respectively, worldwide and 7.2 and 2.1 per 100,000, respectively, in Japan [2, 4]. It is more common in men than in women, with a worldwide age-standardized incidence rate of 9.5 versus 2.4 per 100,000, respectively, and 12.6 versus 2.8 per 100,000, respectively, in Japan [2, 4]. Approximately 20% of patients presenting with invasive urothelial cancer have metastatic or unresectable disease [5]. The gold-standard first-line treatment for patients with locally advanced or metastatic urothelial carcinoma is platinum-based combination chemotherapy (gemcitabine with cisplatin or carboplatin) or methotrexate, vinblastine, doxorubicin, and platinum monotherapy [6–9]. Both atezolizumab and pembrolizumab are approved to treat patients who are ineligible for cisplatin-containing therapy in some countries [10]. There is currently no internationally accepted standard second-line treatment; globally, single-agent paclitaxel and docetaxel are commonly used, and vinflu-nine has been approved in Europe [11–14].

Pembrolizumab is an IgG4 anti-programmed cell death protein 1 (PD-1) humanized antibody that binds to PD-1 and blocks the binding of PD-1 to its ligands, programmed death ligand 1 and programmed death ligand 2 [15]. It was the first immune checkpoint inhibitor that significantly improved overall survival while showing improved tolerability compared with chemotherapy when administered as second-line therapy for patients with platinum-refractory advanced urothelial carcinoma in the open-label, international, phase 3 KEYNOTE-045 trial [16]. Pembrolizumab was approved in Japan in December 2017 to treat patients with radically unresectable urothelial carcinoma who progressed after cancer chemotherapy [17]. The safety and efficacy of pembrolizumab for the treatment of these patients has been shown in clinical studies [16, 18, 19]. Although a large retrospective study was conducted recently [18], the real-world clinical safety and effectiveness of pembrolizumab for this indication is not yet fully confirmed.

This nation-wide, all-case, post-marketing surveillance (PMS) was conducted at the request of Japanese regulatory authorities as a condition of the approval of pembrolizumab. During this study period, pembrolizumab was the only immune checkpoint inhibitor approved for second-line treatment in Japan, where no immune checkpoint inhibitor has been used for first-line treatment. Given the limited number of Japanese patients in the clinical trials leading up to the approval of pembrolizumab, the safety of this drug in the real-world setting was considered unconfirmed. Safety data for pembrolizumab in Japanese patients with lung cancer have been accumulating since approval; however, such data in patients with other cancer types are lacking and currently limited to non-small-cell lung cancer (NSCLC) [20] and melanoma [21]. This PMS aimed to identify factors associated with the safety and effectiveness of pembrolizumab in Japanese patients with unresectable urothelial carcinoma and to confirm the safety and effectiveness of pembrolizumab in the real-world clinical setting by collecting data from a large number (> 1000) of Japanese patients with metastatic urothelial carcinoma.

## Methods

### Study design and treatment

This multicenter, observational, all-case PMS had a 1-year observation period from the start of treatment with pembrolizumab or 30 days after the last pembrolizumab dose for patients with an adverse event (AE). Pembrolizumab monotherapy was initiated at a fixed dose of 200 mg every 3 weeks. Patients from 455 medical institutions were enrolled from 25 December 2017 to 20 April 2018. The survey period ended on 20 April 2020. The data cut-off was 3 March 2021.

This was a company-initiated study, and all data handling was carried out by authors affiliated with the company. This PMS conformed to the provisions of the Declaration of Helsinki and was conducted based on the Good Post-Marketing Study Practice authorized by the Ministry of Health, Labour and Welfare of Japan (Ordinance No. 171, 2004). The ordinance exempts the requirement of ethical review and patients’ informed consent in PMS.

### Patients

All Japanese patients with unresectable urothelial carcinoma who had progressed after at least one regimen of chemotherapy and who had started pembrolizumab monotherapy by 20 April 2018 were included. Only data from patients who were treated at institutions that permitted the use of patient data for this study were included.

### Safety assessment

Treatment-related AEs (TRAEs) were defined as any AE in which a causal relationship to the drug could not be ruled out, including those for which a causal relationship was unknown or unreported. AEs were coded using the Medical Dictionary for Regulatory Activities version 23.1, and graded using the Common Terminology Criteria for Adverse Events version 4.0. AEs of special interest (AEOSI) were defined in accordance with the Japanese Risk Management Plan and included the following: interstitial lung disease (ILD; in patients with pre-existing ILD, onset of pembrolizumab-induced ILD included the worsening or recurrence of ILD within the observation period), colitis/severe diarrhea, hepatic function disorder, renal function disorder (e.g., tubulointerstitial nephritis), endocrinological disorder (e.g., pituitary function disorder, thyroid function disorder, adrenal function disorder), type 1 diabetes mellitus, uveitis, myositis/rhabdomyolysis, pancreatitis, nerve disorders (e.g., Guillain–Barré syndrome), severe skin disorder (e.g., oculomucocutaneous syndrome, erythema multiforme, pemphigoid), encephalitis/meningitis, myasthenia gravis, myocarditis, immune thrombocytopenic purpura, hemolytic anemia, pure red cell aplasia, infusion reaction, and pembrolizumab administration in patients with organ transplant history including a medical history of hematopoietic stem cell transplantation (listed in the Japanese Risk Management Plan as an important identified risk) [22].

### Effectiveness assessment

Tumor response observed during treatment with pembrolizumab was evaluated using Response Evaluation Criteria in Solid Tumors version 1.1. The objective response rate (ORR) was defined as the proportion of patients with complete response (CR) and partial response (PR). The disease control rate (DCR) was defined as the proportion of patients with CR, PR, and stable disease (SD).

### Statistical methods

The target sample size was 500 patients. In the KEYNOTE-045 study, the incidences of hepatic dysfunction, thyroid dysfunction, renal dysfunction, ILD, and colitis/severe diarrhea were 13.2%, 9.4%, 7.1%, 4.1%, and 3.4%, respectively. Assuming an equivalent post-marketing incidence rate, the number of patients necessary to obtain 1% or greater incidence of AEs, including those mentioned above, with 99% confidence was calculated to be 500.

Analysis populations included the safety analysis set and effectiveness analysis set (see Additional file 1). Baseline demographic and clinical characteristics are summarized as mean ± standard deviation for categorical variables and median (range) for continuous variables. Safety data are summarized as the number and percentage of patients experiencing a TRAE or AEOSI. Univariate logistic regression analysis was used to obtain the odds ratio and two-sided 95% confidence interval, with the presence or absence of AEs as the objective variable and background and treatment factors as explanatory variables. Multivariate logistic regression analysis was carried out based on the univariate analysis results. These analyses were conducted to identify background characteristics associated with developing ILD (univariate and multivariate) and hepatic function disorder (univariate). To identify factors associated with the effectiveness of pembrolizumab, the ORR was summarized according to patient subgroups based on baseline demographic and clinical characteristics. Missing values were excluded from the analysis. All statistical analyses were conducted using SAS version 9.4 (SAS Institute; Cary, NC, USA).

## Results

### Patients

A total of 1320 patients were registered by 20 April 2018, and case report forms were collected from 1302 patients. The safety analysis set included 1293 patients, and the effectiveness analysis set included 1136 patients (see Additional file 2).

The median (range) treatment and observation periods were 13.1 (0.1–52.1) and 18.7 (0.1–92.7) weeks, respectively. At 12 months, 79.5% of patients (1028/1293) had discontinued treatment, 20.3% (263/1293) continued treatment, and 0.2% (2/1293) had an unknown treatment status. Among patients who discontinued treatment, the reasons for discontinuation were disease progression, 53.4% (549/1028); death, 26.8% (276/1028); AEs, 21.1% (217/1028); and other, 6.8% (70/1028), where the events of disease progression, death, and AEs recorded in any one patient were counted separately.

Patient baseline demographic and clinical characteristics are summarized in Table 1. Among the 1293 patients in the safety analysis set, the median (range) age was 71 (35–92) years, and most patients were male (75.3%, *n* = 974) and had an Eastern Cooperative Oncology Group performance status (ECOG PS) of 0–1 (87.2%, *n* = 1128). The proportions of patients with 2 and ≥ 3 prior chemotherapy regimens were 30.9% (*n* = 399) and 14.3% (*n* = 185), respectively. The proportions of patients with Bellmunt risk scores of 2 and 3–4 were 29.8% (*n* = 385) and 24.0% (*n* = 310), respectively.

**Table 1:** Baseline demographic and clinical characteristics (safety analysis set)

Characteristic	Number of patients *N* = 1293	%
Age, years		
Median (range)	71 (35–92)	
≥ 65	1006	77.8
≥ 75	445	34.4
Sex		
Male	974	75.3
ECOG PS		
0	696	53.8
1	432	33.4
≥ 2	165	12.8
Time from diagnosis		
< 1 year	315	24.4
≥ 1 year to < 3 years	577	44.6
≥ 3 years	368	28.5
Primary site^a^		
Bladder	692	53.5
Renal pelvis	389	30.1
Ureter	329	25.4
Comorbidity		
Auto-immune disease	31	2.4
ILD	19	1.5
Liver dysfunction	87	6.7
Smoking history		
No	496	38.4
Yes, current	83	6.4
Yes, former	424	32.8
Yes, uncertain	7	0.5
Unknown/blank	283	21.9
Brinkman index^b^		
< 600	666	51.5
≥ 600	253	19.6
Unknown/blank	374	28.9
Hemoglobin < 10 g/dL	452	35.0
Lung metastases	538	41.6
Liver metastases	280	21.7
Prior surgery	1089	84.2
Prior radiation	377	29.2
No. of prior chemotherapies		
Only NAC/adjuvant	29	2.2
1	679	52.5
2	399	30.9
≥ 3	185	14.3
Duration from last dose		
< 3 months	873	67.5
≥ 3 months	404	31.2
Number of Bellmunt risk scores^c^		
0	156	12.1
1	425	32.9
2	385	29.8
3–4	310	24.0

*ECOG PS* Eastern Cooperative Oncology Group performance status, *NAC* neoadjuvant chemotherapy, *ILD* interstitial lung disease

^a^Includes some overlapped cases

^b^Estimate of the cumulative smoking dose: number of cigarettes smoked per day × total number of years smoking

^c^Based on the presence of the following factors: ECOG PS ≥ 1, hemoglobin < 10 g/dL, duration from last dose < 3 months, presence of liver metastases

### Safety

**Fig. 1:**
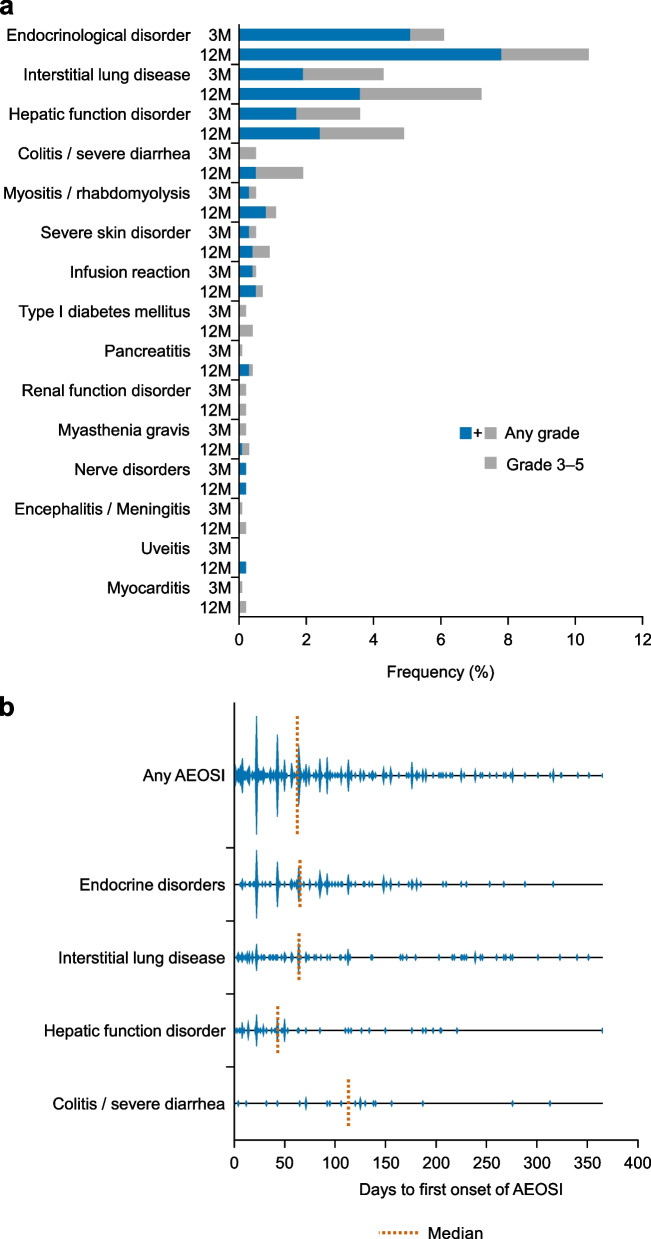
AEOSI incidence at 3 and 12 months (**a**) and AEOSI onset occurring in ≥ 20 patients (**b**). *AEOSI* adverse event of special interest, *M* months

**Fig. 2:**
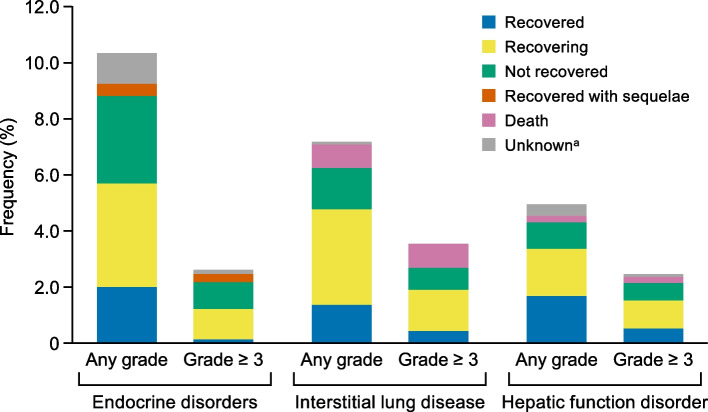
Outcomes of adverse events of special interest. ^a^“Unknown” does not include “death”

The incidence of TRAEs and AEOSI at 3 months and 1 year is shown in Table 2. At 12 months, the incidence of TRAEs was 53.8% (*n* = 696) and that of AEOSI was 25.0% (*n* = 323). Grade 5 TRAEs occurred in 83 patients (6.4%), 47 (3.6%) of which were due to disease progression. Grade 5 AEOSI were reported in 1.4% (*n* = 18) of patients (Table 3). The most frequent lethal event was ILD, which occurred in 11 patients (0.9%). AEOSI by grade and time of occurrence are summarized in Fig. 1a. At 3 months and 12 months, the most frequent AEOSI of any grade were endocrinological disorder (10.4%, *n* = 134; Grade 3–5, 2.6%), ILD (7.2%, *n* = 93; Grade 3–5, 3.6%), hepatic function disorder (4.9%, *n* = 64; Grade 3–5, 2.5%), and colitis/severe diarrhea (any grade: 1.9% *n* = 25; Grade 3–5, 1.4%). A violin-plot of AEOSI onset is shown in Fig. 1b. Most AEOSI appeared within the first three dosing cycles (63 days), which was notable for hepatic and endocrine disorders. Both early- and late-onset (i.e., beyond 200 days) ILD occurred, which contrasted with the onset of the other AEOSIs, particularly for colitis/severe diarrhea, which that had few late-onset cases. The outcomes of AEOSI are summarized in Fig. 2. No deaths were reported among patients with endocrinological disorder, but some patients did not recover or recovered with sequelae. Among patients who received corticosteroids for the treatment of endocrine disorder, the dose of administered corticosteroid was low (median: 945 mg) and the treatment period was long (median: 138 days) (see Additional file 3). There were no patients with organ transplant history who received pembrolizumab in this surveillance.

**Table 2:** Incidence of TRAEs and AEOSI (safety analysis set)

	**0–3 months**		**0–12 months**	
	***N* = 1293**	**%**	***N* = 1293**	**%**
TRAE^a^				
Any grade	487	37.7	696	53.8
Grade 3–5	183	14.2	293	22.7
AEOSI^a^				
Any grade	199	15.4	323	25.0
Grade 3–5	86	6.7	141	10.9
Grade 5	14	1.1	18	1.4

^a^Includes some overlapped cases

*AE* adverse event, *AEOSI* adverse event of special interest, *TRAE* treatment-related adverse event

**Table 3:** Grade 5 AEOSI

	**AEOSI**	
	***n* = 18**	**%**
Total	18	1.4
Interstitial lung disease	11	0.9
Hepatic function disorder	3	0.2
Colitis / severe diarrhea	1	0.1
Renal function disorder (tubulointerstitial nephritis, etc.)	1	0.1
Encephalitis / meningitis	1	0.1
Myasthenia gravis	1	0.1

*AEOSI* adverse event of special interest

**Fig. 3:**
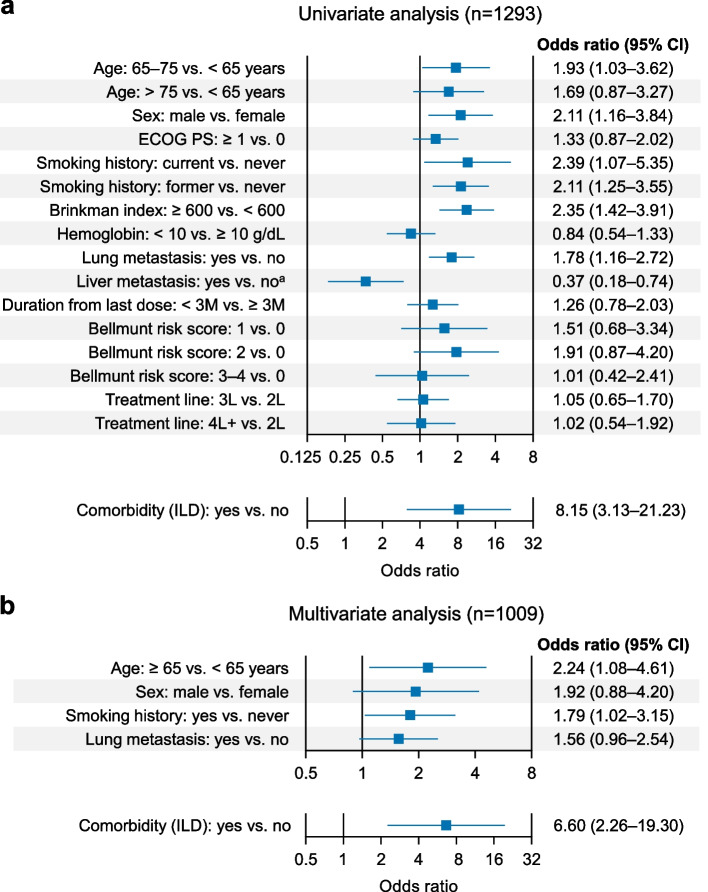
Background characteristics associated with the development of ILD by univariate (**a**) and multivariate (**b**) analysis. ^a^Liver metastasis was not included in the multivariate analysis because the clinical relevance is uncertain. *CI* confidence interval, *ECOG PS* Eastern Cooperative Oncology Group performance status, *ILD* interstitial lung disease, *L* line, *M* months

As for clinical factors associated with AEOSI, univariate analysis showed that the risk of ILD was higher in the elderly (odds ratio 1.93 in those aged 65–75 years), male patients (odds ratio 2.11), and those with a history of smoking (odds ratio 2.39 in current smokers, 2.11 in former smokers), lung metastasis (odds ratio 1.78), and ILD as a comorbidity (odds ratio 8.15) (Fig. 3a). The risk of ILD was lower in patients with liver metastasis (odds ratio 0.37) (Fig. 3a). Multivariate analysis demonstrated that the risk of developing ILD was almost seven times greater in patients with a comorbidity of ILD at baseline (odds ratio 6.60) and approximately twice as high in patients aged ≥ 65 years (odds ratio 2.24) and patients with smoking history (odds ratio 1.79) (Fig. 3b).

Regarding other frequent AEOSI, univariate analysis showed that hepatic function disorder was less likely to occur in men (odds ratio 0.53). Endocrinological disorders were more likely to occur in patients with a body mass index ≥ 25 kg/m^2^ (odds ratio 1.55) and less likely to occur in patients with hemoglobin < 10 g/dL (odds ratio 0.44) or ECOG PS ≥ 1 (odds ratio 0.60).

### Effectiveness

**Fig. 4:**
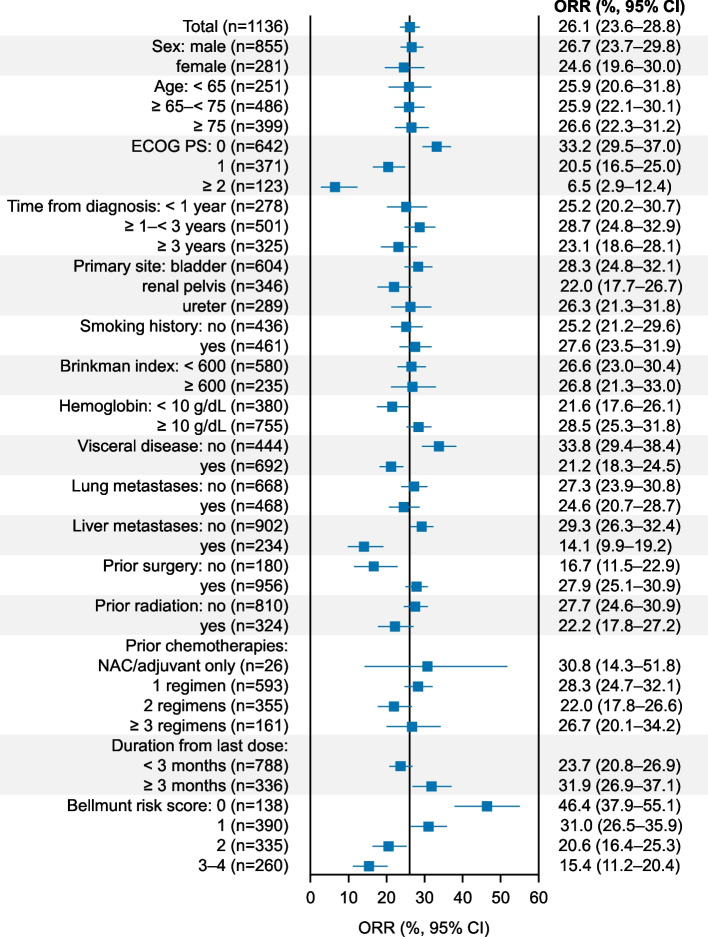
Forest plot for ORR by background patient characteristics (effectiveness analysis set). *CI* confidence interval, *ECOG PS* Eastern Cooperative Oncology Group performance status, *NAC* neoadjuvant chemotherapy, *ORR* objective response rate

**Fig. 5:**
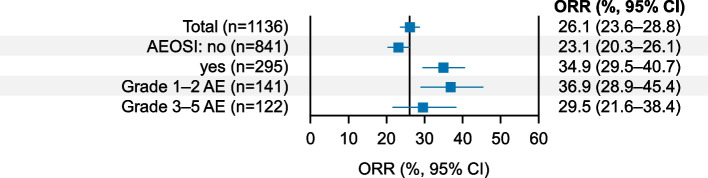
Forest plot for ORR by AEOSI (effectiveness analysis set). *AE* adverse event, *AEOSI* adverse event of special interest, *CI* confidence interval, *ORR* objective response rate

In the effectiveness analysis set (*n* = 1136), the tumor response was CR in 64 patients (5.6%), PR in 233 patients (20.5%), SD in 279 patients (24.6%), and progressive disease in 560 patients (49.3%). The ORR was 26.1% and the DCR was 50.7%. The ORR by background patient characteristics is shown in Fig. 4. The ORR was 46.4% in patients with a Bellmunt risk score of 0 and decreased as the Bellmunt risk score increased. The ORR was relatively low in patients with characteristics including an ECOG PS of ≥ 2, liver metastases, and no prior surgery. ORRs in patients with and without AEOSI were 34.9% and 23.1%, respectively, and 36.9% in patients with Grade 1–2 AEOSI (Fig. 5). There was no notable difference in the ORR according to concomitant corticosteroid use (without concomitant corticosteroid use, 25.4% [215/848]; with concomitant corticosteroid use, 28.8% [82/285]).

## Discussion

Pembrolizumab is the first immune checkpoint inhibitor approved for the second-line treatment of metastatic urothelial carcinoma and is the only drug approved in Japan for second- or later-line treatment. Its efficacy and safety have been confirmed in the phase 3 KEYNOTE-045 trial [16]. However, it is important to evaluate the safety and effectiveness of pembrolizumab in actual clinical practice, where patients with different characteristics, including those with various complications and poor ECOG PS, are treated. The findings of this survey, which evaluated the safety and effectiveness of pembrolizumab as second-line treatment for metastatic urothelial carcinoma in more than 1000 real-world patients, are of great significance. Of note, the original target sample size for this survey was 500 patients to detect AEOSI with a frequency of 1%, but more than 1300 patients were enrolled, exceeding our expectation. This all-case surveillance enrolled patients with various background characteristics: 12.8% of patients had an ECOG PS ≥ 2, which was higher than that reported in the KEYNOTE-045 trial (0.7%) [16], and 35.0% of patients had a low hemoglobin level (< 10 g/dL). The proportion of heavily treated patients (≥ 2 prior regimens) was higher than that in the KEYNOTE-045 trial (45.2% vs. 20.4%, respectively) [16]. However, the safety profile and AEOSI rates were comparable to that reported in the KEYNOTE-045 trial, and this finding is important because it is the first to demonstrate the safety of pembrolizumab in a large real-world data set.

No unknown safety concerns were raised in this PMS, and the AE profile was generally consistent with that of previous reports. The most frequent AEOSI was endocrine disorder, followed by ILD, hepatic function disorder, and colitis/severe diarrhea. Other AEOSIs were relatively infrequent, but some of them were serious (e.g., Grade 5 renal function disorder, encephalitis/meningitis, and myasthenia gravis). Although the frequency of renal function disorder was low (0.2%), many patients with urothelial cancer have poor renal function, and platinum-based agents often cause renal disorders.

As for the timing of AEOSI, most appeared within the first three dosing cycles (63 days), which was notable for hepatic and endocrine disorders, although the violin-plot revealed that both early-onset and late-onset ILD was observed. These findings suggest that when treating these patients, special attention should be paid to the potential development of AEOSI including rare ones, and constant patient monitoring is also needed from the start of treatment with pembrolizumab.

A previous PMS of pembrolizumab in patients with advanced NSCLC reported that the most frequent AEOSI was ILD (any grade: 12.2%), followed by endocrine disorders (8.5%), liver dysfunction (5.2%), and colitis/severe diarrhea (2.4%) [20], while another PMS in patients with melanoma reported that the most frequent AEOSI were endocrine disorder (any grade: 9.9%), liver dysfunction (6.1%), ILD (5.1%), and colitis/severe diarrhea (3.1%) [21]. These trends of frequent AEOSI were similar to those observed in patients with metastatic urothelial carcinoma. However, a previous study showed that there were some differences in the AE incidence among patients with various cancer types who were treated with immune checkpoint inhibitors [23]. The incidence of ILD in patients with metastatic urothelial carcinoma (7.2%) was lower than that in patients with NSCLC, but was much higher than that in patients with melanoma. These differences of ILD incidence might be due to differences in smoking rates between the different study populations; the smoking rate was especially high in patients with NSCLC (77.8%). Other differences in AE incidence between the different cancer types may also be a result of differences in tumor/patient characteristics, so it would be meaningful to assess the safety of a single drug in patients with different tumor types.

We investigated risk factors associated with the three most frequent AEOSI (ILD, hepatic function disorder, and endocrine disorder). For hepatic function disorder and endocrine disorder, we could not find clinically meaningful risk factors. Age (≥ 65 years), smoking history, and ILD comorbidity were identified as risk factors for the development of ILD. In particular, ILD comorbidity was the strongest risk factor (odds ratio 6.60). A recent PMS of pembrolizumab in patients with NSCLC reported a higher likelihood of experiencing ILD onset or deterioration as an AE in the first-line setting with current/former smokers, those with a history of pre-existing ILD, and those with a history of or current neoplasms (except lung cancer) [20]. Therefore, special attention is necessary for such patients with smoking history or ILD comorbidity, regardless of the tumor type. Of note, liver metastasis was found to be associated with a lower risk of developing ILD in the univariate analysis. However, considering the uncertain clinical relevance of this finding, liver metastasis was not included in the multivariate analysis. This does not suggest that liver metastasis is a protective factor for ILD because liver metastasis is a poor prognostic factor for urothelial carcinoma. Most likely, the short treatment duration in patients with liver metastasis may reduce the onset of ILD.

The ORR of pembrolizumab was 26.1% (CR: 5.6%, PR: 20.5%) in this study, and this result is consistent with previous reports [16, 18, 19]. Bellmunt risk score (ECOG PS > 1, hemoglobin < 10 g/dL, duration from last dose < 3 months, and presence of liver metastasis) is a prognostic factor in conventional second-line treatment [24]. The results of this surveillance also showed that the ORR in patients with Bellmunt risk score 0 was the highest (46.4%) compared with patients with other Bellmunt risk scores and decreased as the Bellmunt risk scores increased. The ORR was > 30% in patients with ECOG PS ≤ 1 or no visceral metastasis. This is consistent with a previous study that investigated prognosis stratified by PS, hemoglobin, metastasis sites, and neutrophil-to-lymphocyte ratio [25]. Several studies reported that the neutrophil-to-lymphocyte ratio was also a predictor of pembrolizumab efficacy in urothelial carcinoma [25–27].

In a clinical study of pembrolizumab for urothelial carcinoma, patients with immune-related AEs (irAEs) had better outcomes than those without irAEs [28]. Although a relationship between AEOSI (i.e., immune-related AEs) and effectiveness cannot be confirmed, we found that the ORR tended to be better in patients with Grade 1–2 AEOSI than in patients without AEOSI. The timing of irAEs and assessment of effectiveness was not considered in this analysis, but it should be noted that patients with better outcomes tend to receive a longer course of treatment, which would increase the risk of irAE occurrence and may provide an explanation for this finding.

This study had several limitations. The duration of treatment and follow-up was limited. The patient population had a variable treatment history and there were some missing data. Moreover, safety and effectiveness were not centrally assessed, and the timing of the effectiveness evaluation was not specified in advance because this was an investigation of real-world effectiveness of pembrolizumab. Finally, this study evaluated only ORR as a measure of effectiveness. A recent real-world study of pembrolizumab in patients with advanced, unresectable, or metastatic urothelial carcinoma reported overall survival as a measure of effectiveness [29]. In that study, overall survival was longer in those treated with pembrolizumab versus those treated with conventional chemotherapy, regardless of their background characteristics.

## Conclusions

In this PMS, the safety and effectiveness of pembrolizumab were confirmed in Japanese patients with unresectable urothelial carcinoma in the real-world setting and no new safety signal was raised. ILD was the most lethal event and age (≥ 65 years), smoking history, and ILD comorbidity were identified as risk factors for the development of ILD in patients treated with pembrolizumab.

## Supplementary Information

**Additional file 1: **Safety analysis set and effectiveness analysis set criteria.

**Additional file 2: **Patient disposition.

**Additional file 3: **Corticosteroid use for AEOSI.

## Data Availability

The data sets analyzed during this post-marketing surveillance are not available because data sharing with third parties is not permitted per the contract with all study sites or the patients. Please contact MSD K.K. (https://www.msd.co.jp) for inquiries about access to the data set used in this post-marketing surveillance.

## Abbreviations

AE: Adverse event
AEOSI: Adverse event of special interest
CR: Complete response
DCR: Disease control rate
ECOG PS: Eastern Cooperative Oncology Group performance status
ILD: Interstitial lung disease
irAEs: Immune-related AEs
L: Line
M: Months
NSCLC: Non-small-cell lung cancer
ORR: Objective response rate
PD-1: Programmed cell death protein 1
PMS: Post-marketing surveillance
PR: Partial response
SD: Stable disease
TRAE: Treatment-related adverse event
