# Supplementary material for: Safety and effectiveness of pembrolizumab monotherapy in Japanese patients with unresectable urothelial carcinoma: a nation-wide post-marketing surveillance
Source: BMC Cancer. 2023 Jun 20;23:565. doi: 10.1186/s12885-023-10930-2 (PMC10280973; doi:10.1186/s12885-023-10930-2)
Supplement: Supplementary file 1 — Additional file 1: Safety analysis set and effectiveness analysis set criteria. [file 12885_2023_10930_MOESM1_ESM.pdf]

Additional file 1

Safety analysis set and effectiveness analysis set criteria

Patients were excluded from the safety analysis set if they were outside the registration period, treated with pembrolizumab outside the study period, in violation of the contract, attended an initial visit but no subsequent visits, were missing clear information on whether an adverse event occurred, did not receive any doses of pembrolizumab, had no background information, had a history of prior pembrolizumab use, or if safety reinvestigation was not possible. Duplicated entries were also excluded.

Patients with unreported effectiveness endpoints, cases evaluated using a criterion other than Response Evaluation Criteria in Solid Tumors, and those treated with pembrolizumab for an indication other than "radically unresectable urothelial carcinoma that progressed after cancer chemotherapy", treated with an off-label regimen of pembrolizumab, and who received another anticancer agent during their pembrolizumab treatment were excluded from the effectiveness analysis set.
