# Supplementary material for: Safety and effectiveness of pembrolizumab monotherapy in Japanese patients with unresectable urothelial carcinoma: a nation-wide post-marketing surveillance
Source: BMC Cancer. 2023 Jun 20;23:565. doi: 10.1186/s12885-023-10930-2 (PMC10280973; doi:10.1186/s12885-023-10930-2)
Supplement: Supplementary file 2 — Additional file 2: Patient disposition. [file 12885_2023_10930_MOESM2_ESM.pdf]

Additional file 2. Patient disposition

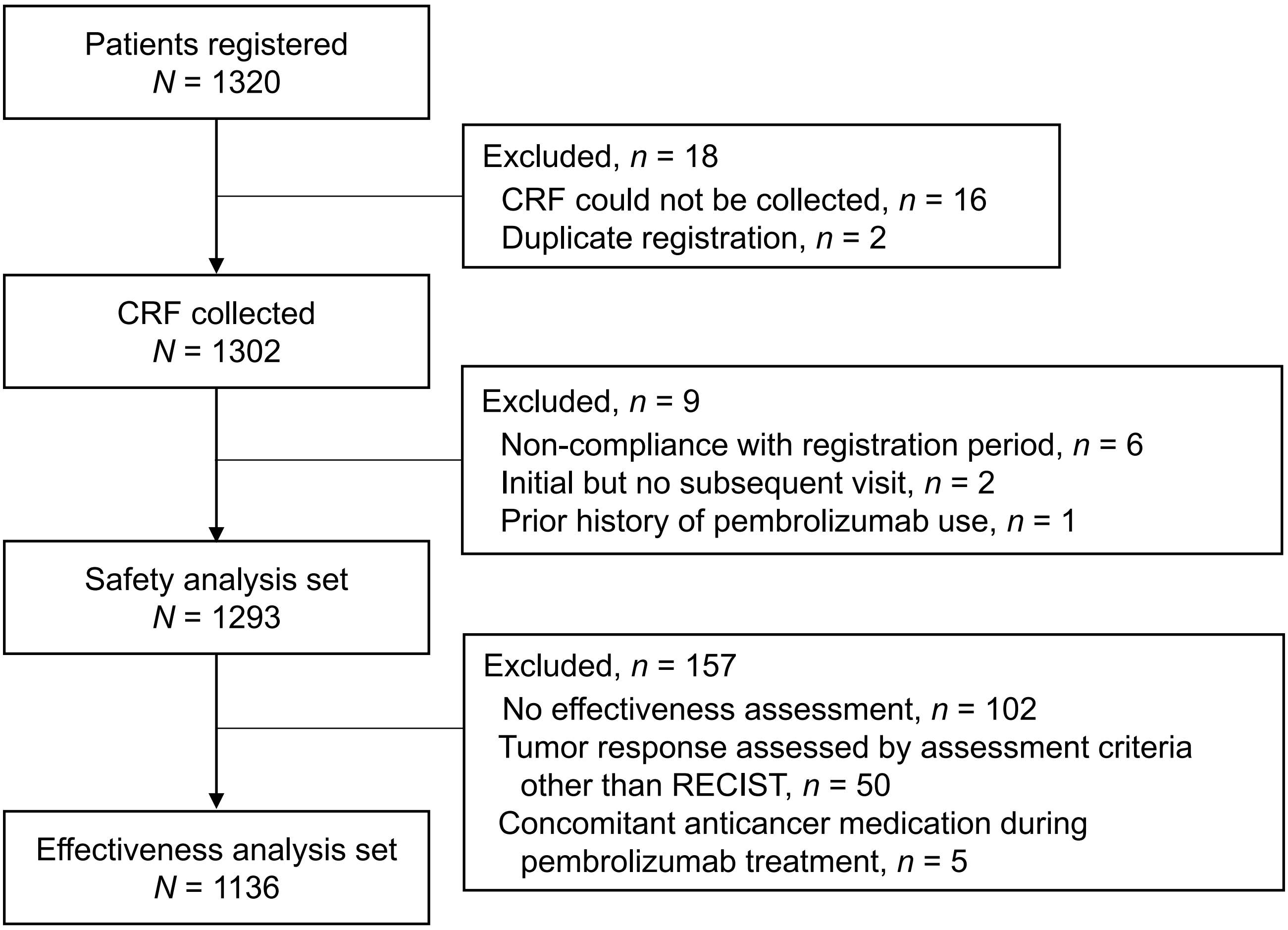

CRF case report form, *RECIST* Response Evaluation Criteria in Solid Tumours.
