# Supplementary material for: Safety and effectiveness of pembrolizumab monotherapy in Japanese patients with unresectable urothelial carcinoma: a nation-wide post-marketing surveillance
Source: BMC Cancer. 2023 Jun 20;23:565. doi: 10.1186/s12885-023-10930-2 (PMC10280973; doi:10.1186/s12885-023-10930-2)
Supplement: Supplementary file 3 — Additional file 3: Corticosteroid use for AEOSI. [file 12885_2023_10930_MOESM3_ESM.pdf]

Additional file 3. Corticosteroid use for AEOSI

	Endocrine disorder	Interstitial lung disease	Hepatic function disorder
No. of patients with AEOSI, *N*	134	93	64
Corticosteroid use, *n* (%)	28 (20.9)	60 (64.5)	16 (25.0)
Corticosteroid pulse use, *n* (%)	0 (0.0)	22 (23.7)	3 (4.7)
Total dose, *n*	25	53	15
Median dose (min–max), mg	945 (40–3270)	2095 (20–30,000)	2167 (163–16,223)
Median duration (min–max)[a], days	138 (6–351)	39 (1–376)	55 (3–316)

[a]Patients continued corticosteroid use after the observation period but could not be followed

AEOSI adverse event of special interest
